# Efficacy and Safety of Therapies for Acute Ischemic Stroke in China: A Network Meta-Analysis of 13289 Patients from 145 Randomized Controlled Trials

**DOI:** 10.1371/journal.pone.0088440

**Published:** 2014-02-13

**Authors:** Bowen Yang, Jingpu Shi, Xin Chen, Bing Ma, Hao Sun

**Affiliations:** Department of Clinical Epidemiology, Institute of Cardiovascular Diseases and Center of Evidence Based Medicine, The First Affiliated Hospital, China Medical University, Shenyang, China; National Taiwan University, Taiwan

## Abstract

**Background:**

Many of these therapies have been compared against placebos, but have not been directly compared against each other. To evaluate the efficacy and safety of several commonly used drugs for AIS directly or indirectly.

**Methods:**

A systematic literature review was performed to identify randomized controlled trials (RCTs) published prior to April 2013 for AIS therapies. The primary outcome measures were the National Institutes of Health Stroke Scale (NIHSS) scores and the clinical effective rate. A fixed-effects meta-analysis and meta-regression are performed; lastly, performed a mixed treatment comparison was performed through the Bayesian methods.

**Results:**

Outcome of Efficacy of therapies for acute ischemic stroke are as followed: All of the therapies mentioned above yielded results a more effective result than placebo, Sodium ozagrel (RR 3.86, 95%CI 3.18–4.61); Sodium ozagrel + edaravone (RR 9.60, 95%CI 7.04–13.06); Edaravone (RR 4.07, 95%CI 3.30–5.01); Edaravone + Kininogenase (RR 15.33, 95%CI 10.03–23.05). The significant difference in efficacy between edaravone monotherapy and Sodium ozagrel + edaravone was evident (RR 0.43, 95%CI 0.08–0.61) and was also significant between efficacy of edaravone + Kininogenase and Sodium ozagrel (RR 4.00, 95%CI 2.47–6.24). The differences between the risk and benefit were not significant when comparing Sodium ozagrel and edaravone or edaravone + Kininogenase and Sodium ozagrel + Edaravone for AIS. Outcome of the defect of neurological function: Placebo served a significant difference in treating the defects of neurological function compared with Sodium ozagrel (WMD = −3.11, 95%CI −4.43 to −1.79), Sodium ozagrel + edaravone (WMD = −6.25, 95%CI −7.96 to −4.54) and Edaravone + Kininogenase (WMD =  −3.47, 95%CI −5.73 to −1.21).

**Conclusions:**

It provides that the efficacy of edaravone monotherapy in treatment was not more effective than Sodium ozagrel + edaravone.The efficacy of edaravone + Kininogenase monotherapy in treatment was more effective than Sodium ozagrel. Edaravone + Kininogenase and Sodium ozagrel + Edaravone appeared the most effective treatments. And Sodium ozagrel, Sodium ozagrel + edaravone, Edaravone + Kininogenase can improve the nerve dysfunction.

## Introduction

Stroke is the second most common cause of death and the major cause of disability worldwide, preceded only by heart attack. The burden of stroke will increase greatly in the next 20 years, especially in developing countries, due to the aging of population [1]. About 80% of all acute strokes are caused by cerebral infarction, usually resulting from thrombotic or embolic occlusion of a cerebral artery [2]. In addition, China must face the greatest number of cases of stroke due to its 1.34 billion population accounted for as of 2011 [3]. The epidemiological data shows that from 1984 to 2004 the incidence of ischemic stroke as the most common subtype, representing about 80% of all strokes, and has increased by 8.7% per year even though the incidence of hemorrhagic stroke decreased by 1.7% every year in China [3]. Guideline from the American Heart Association/American Stroke Association for the patients' early management of acute ischemic stroke remains multifaceted, and includes several aspects of care that have not been tested in clinical trials [4]. Although data from some experimental and human studies have suggested that edaravone and Sodium ozagrel, the neuroprotective agent, may be beneficial for people with acute ischemic stroke, It has been widely used in China to treat stroke [5], [6], the comparisons of these drugs or combined treatments were unclear.

Network meta-analysis is an extension of traditional meta-analysis as well as a method that synthesizes available evidence to allow for simultaneous comparisons of different treatment options that lack direct head-to-head evaluations [7]–[10]. Individual pair-wise studies are ‘linked’ to create a network of studies on which statistical inference is based. When the network consists of a mixture of direct and indirect evidence with comparable studies and patient characteristics, the relative treatment effect of drug B vs. drug C may be indirectly estimated by comparing studies of drug A vs. drug B and drug A vs. drug C (dbc = dac _ dab) [10], [11]. The value of a network meta-analysis is that it can include both direct and indirect evidence, and it preserves the strength of randomization within individual RCTs. However, the method has limitations as it is based on a model simulation, and the method could result in biased estimates, if there are systematic differences across comparisons.

## Methods

### 2.1 Search strategy

We searched MEDLINE, EMBASE, CNKI, PubMed, The Cochrane Central Register of Controlled Trials and The Cochrane Library prior to April 2013. The authors were contacted for further information regarding unpublished trials and reports found in published databases. Search terms included MeSH: acute ischemic stroke, AIS AND Edaravone OR Sodium ozagrel OR edaravone combined with Kininogenase OR Sodium ozagrel combined with edaravone AND randomized controlled trial (RCT). We also identified studies through manual searches of article references. No search limits were applied. Table S1 describes the literature search methods and restrictions applied to our search. We evaluated comparability of effect modifiers and potential heterogeneity between studies by abstracting information about base-line patient characteristics and trial characteristics including drug dosing regimens.

### 2.2 Study selection

For each literature search, all of the titles and abstracts were thoroughly reviewed and deemed to meet the following:

Inclusion criteria: (1) Original RCT evaluating a AIS therapy, (2) Previously untreated patient population, (3) AIS, including all grades and/or grade three to grade four, (4) reports of odds radio (OR), 95% confidence intervals (CIs) or data were available, (5) Entire study is available for review, (6) patients of which are Chinese. (7) Limited in ‘human’ study with no language restriction.

The criteria of neurological deficit score were adopted based on the Modified Edinburgh–Scandinavian-Stroke Scale, a nation wide accepted scoring system recommended at the Second, and revised at the Fourth National Cerebrovascular Disease Conference in China [12]. The assessment of the clinical efficacy rate was conducted in accordance with the reduction in the scores of basic nervous functional deficits and disability degree as following :Recovery-The functional deficit scores were decreased up to 91–100%, and disability degree was at grade 0; Remarkable Improvement-The scores of functional deficit were decreased at 46–90%, and disability degree was at the grade1–3; improvement—the scores of functional deficit were decreased at 18–45%; no change—the scores of functional deficit were decreased or increased at about 17%; deterioration—the scores of functional deficit were increased over 18% and death.

### 2.3 Data extraction

Two authors assessed the methodological quality of the trials and extracted the data. Complete articles were used where possible. Disagreements were resolved with the assistance of a third reviewer.

### 2.4 Methodological quality of studies

Two authors (Yang and Chen) extracted review characteristics, the benefits and safety results, and they assessed the quality of the reviews using the Assessment of Multiple Systematic Reviews (AMSTAR) quality-assessment instrument [13].

### 2.5 Statistical analysis

Data were gathered from all the publications into a computerized spreadsheet (Microsoft Excel), and then turned into a traditional meta-analysis using fixed and random effects models by pooled risk ratio (RR) using STATA 10.0. The 95% CIs were calculated. This study had two components. First, traditional direct meta-analyses of therapies versus placebo, ozagrel versus ozagrel + edaravone and edaravone + kalliclingenase were preformed. Second, these direct meta-analyses were combined using network meta-analysis. A separate spreadsheet was produced for the 145 individual two-by-two comparisons, the logarithm of the individual relative ratio, and SE (calculated by the Mantel and Haenszel method), and used for the network meta-analysis. Network meta-analysis is a relatively new technique that can be used to indirectly compare the risk or benefit associated with randomly allocated treatments that have not been directly tested against each other [14].

The trials had to be conducted with populations of similar age and disease profile sharing a common treatment or placebo arm [15], [16]. For this instance, a network meta-analysis to estimate the baseline risk or benefit associated with the administration of ozagrel versus edaravone, ozagrel + edaravone versus edaravone + Kininogenase and edaravone versus ozagrel + edaravone.

National Institutes of Health Stroke Scale (NIHSS) scores were extracted as mean±s.d. When unreported, missing mean differences and s.d.'s were estimated based on reported CIs, and the authors of the study were contacted if necessary. The s.e.m. was transformed into s.d. weighted mean difference (WMD) and 95% CIs, in the defect of neurological function were calculated for all eligible studies in the network meta-analysis and combined using fixed or random-effects models where appropriate.

Later, a network meta-analysis was performed using the one-line program published by Lumley [17], We used WinBUGS 1.4.3 (Bayesian inference Using Gibbs Sampling for Microsoft Windows), a freely available statistical software package that uses Markov Chain Monte Carlo (MCMC) simulation to conduct the analysis [18] (programming language R, version 2.15.3).

### 2.6 Sensitivity analyses

During model development, we fit separate fixed effect and random effect models, in which the differences in scale parameters of each curve were modeled on the log hazard scale. For the fixed effect models, we assumed that the true treatment effect of a given therapy is the same across all trials included in the comparison network. For the random effect models, we assumed that the true treatment effect of a given therapy is similar, but not the same, across all trials included in the comparison network and is exchangeable between studies. We use heterogeneity parameters in our random effect models, which is the scale parameter. We assigned a non-informative bivariate normal distribution to the mean of scale parameters of the baseline treatment in each trial, and also to the difference in scale parameters between survival curves relative to the baseline in each study. We assigned a non-informative Wishart distribution to the variance of the difference in scale parameters between studies, and assumed constant heterogeneity across treatment comparisons. We used vague initial values to analyze both models using two chains and 80,000 iterations after a burn-in of 40,000 iterations. We evaluated convergence by monitoring trace plots, autocorrelations, and Monte Carlo error which describes the variability of each estimate due to the simulations. We evaluated goodness-of-fit of the Weibull and log–logistic models by visually evaluating linearity of diagnostic data plots and comparing the deviance information criteria (DIC) between models, where smaller DIC values indicate a better fitting model [19]. We also used DIC to evaluate the goodness-of-fit of fixed effect versus random effect models. We conducted sensitivity analysis by varying the initial parameter values.

### 2.7 Inconsistency

Some authors have proposed ways to assess consistency directly from fitting consistency models. The method we want to use for testing inconsistency is ‘Loop inconsistency’. We call this loop inconsistency and depict it by drawing edges in different line styles. Loop inconsistency can arise only from at least three separate sets of studies making different comparisons (for example, ‘B–A’, ‘C–A’ and ‘C–B’ studies). And the model motivated primarily by loop inconsistency is described by Lu and Ades (2006). In this model, one inconsistency parameter is added for each independent closed loop in the evidence network (not including loops created only by multi-arm trials) [20]. The inconsistency of the network was determined by use of Code for Bayesian analysis in WinBUGS and was determined by use of inconsistency factors [21].

## Results

### 3.1 Search results

The search included a total of 1206 citations (**Figure S1**); screened each title and abstract, and excluded 737 papers. The remaining 296 papers were examined as potentially relevant RCTs. 192 papers met the inclusion criteria: 46 were later excluded, and 145 RCTs were included across the three meta-analyses. Most of the studies were performed in PubMed, EMBASE and CNKI and predominantly published in Chinese and English (84.1% CNKI n = 122, 13.1% PubMed n = 19, and 2.8% EMBASE n = 4).

### 3.2 Outcome of Efficacy of therapies for acute ischemic stroke

**Figure S2** shows the results of the network meta-analysis. According to these data, Sodium ozagrel combined with edaravone was more effective treatment for AIS than edaravone (RR 0.43, 95%CI 0.08–0.61), also was significant between efficacy of edaravone combined with Kininogenase and Sodium ozagrel (RR 4.00, 95%CI 2.47–6.24). Since the network meta-analysis technique does not pre-specify the standard, it only compares and calculates the interrelations across all agents, the referent agent can be changed to placebo. When this was done, the rank ordering of the agents according to their propensity to be associated with onset of AIS remained the same. Relative ratios did not differ between an edaravone with Sodium ozagrel (1.06, 0.80–1.36), or between an edaravone combined with Kininogenase and Sodium ozagrel combined with edaravone (1.63, 0.97–2.66) (**Table 1**). **Figure S3** is an example of the graph yield from the five-way network meta-analysis procedure for the outcome of AIS.

**Table 1:** Findings of Network Meta-Analyses, Showing the OR Comparing Drugs (95% Credible Interval).

Placebo	3.86	9.60	4.07	15.33
	3.18–4.61	7.04–13.06	3.30–5.01	10.03–23.05
1.238	Sodium ozagrel	2.4888	1.0633	4.00
1.213–1.264		1.90–3.19	0.80–1.36	2.47–6.24
0.83	0.832	Sodium ozagrel and edaravone	0.43	1.633
0.79–0.88	0.80–0.87		0.30–0.61	0.97–2.66
0.73	——	——	Edaravone	3.77
0.70–0.76				2.58–5.44
0.76	——	——	0.77	Edaravone and Kininogenase
0.65–0.90			0.72–0.82	

Comparisons between drugs should be read from left to right, and the estimate is in the cell in common between the column-defining treatment and the row-defining treatment. For both outcomes, ORs <1 favor the column-defining treatment. To obtain ORs for comparisons in the opposite direction, reciprocals should be taken. OR indicates odds ratio.

### 3.3 Outcome of the defect of neurological function

Placebo had significant differences in treating the defect of neurological function compared with Sodium ozagrel (WMD = −3.11, 95%CI −4.43 to −1.79), Sodium ozagrel combined with edaravone (WMD = −6.25, 95%CI −7.96 to −4.54) and Edaravone combined with Kininogenase (WMD = −3.47, 95%CI −5.73 to −1.21). (**Figure S4**)

Research indicated reporting quality of studies varied. (**Table S2**) All of studies reported how randomization sequence was generated in their primary publication. Eleven studies reported on how allocation to groups was concealed. Most of the studies reported on loss-to-follow up and only four studies reported that the primary results were based on a per-protocol analysis rather than intent to treat. Fifty-three studies reported on at least one specific group being blinded in the trial, typically patients and caregivers.

The results of indirect comparison were highly coincident using the consistency checking. (**Figure S5**) Each treatment data is in the form of triangle loop. Confidence intervals of each ring are intersected with the blank value, the result shows that there is no significant difference between direct and indirect result, namely consistency is good.

### 3.4 Sensitivity analyses

The convergence diagnostic tests showed that the MCMC algorithm reached adequate equilibrium after 80,000 iterations, indicating that the estimated results are derived from an appropriate posterior distribution. The model was compatible to the data with stronger linearity in the graphical diagnostic plots and it had lower DIC values overall. The fixed effect model demonstrated better convergence, smaller standard deviation and Monte Carlo error, narrower credible intervals. It was a better fit to the data with a DIC value of 1489.1, compared to a DIC of 1598 for the random effect model. The fixed effect model may be more relevant in this study due to minimal heterogeneity between the trials in the comparison network.

## Discussion

We conducted a network meta-analysis using a Bayesian analytic framework applied to data abstracted from the RCTs in order to simultaneously compare therapies that have not previously been directly compared. These results summaries experience of the treatment of acute cerebral stroke in short-term clinical trials, incorporating both direct and indirect comparisons of agents, including those that have never been compared directly (i.e. edaravone versus Sodium ozagrel combined with edaravone). The network meta-analysis technique overcomes the significant heterogeneity in traditional meta-analysis for several drug classes versus all other treatment of acute cerebral stroke in short-term clinical trials. Due to the reason that it can attribute risk across all classes of initial treatment of acute cerebral stroke in short-term clinical trials, rather than being restricted to comparisons of one class versus all other classes. The findings of this network meta-analysis are robust, in terms of both the low estimate of incoherence within the model itself, and in sensitivity analyses.

The present study is robust because the prospective design should eliminate selection bias and recall bias. Furthermore, all of the studies included in our network meta- analysis were randomized controlled trails which were the most ideal evidences and all of them were assessed by AMSTAR quality-assessment instrument. Otherwise, there was no evidence of publication bias and our sensitivity analysis showed no change in the statistical significance of the pooled estimate. Therefore, the outcome of present study was convincible.

Edaravone inhibits lipid peroxidation by scavenging free radicals, and exerts neuroprotective effects by inhibiting oxidative damage to the Cerebrovascular endothelium and brain cells [22], [23]. In clinical terms, the efficacy of edaravone was demonstrated in a randomized double-blind placebo-controlled trial, in which the difference in mRS grades was significant at discharge within or at 3 months after stroke onset. A particularly remarkable efficacy was noted within 24 h from stroke onset [24]. The efficacy and safety of ozagrel have been demonstrated in a randomized double-blind placebo-controlled trial, where the differences in the improvement of neurological symptoms were significant compared to the placebo [25]. Kininogenase can selectively dilate ischemic vascular, promote angiogenesis, thereby increasing the ischemic brain tissue blood flow, improve cerebral microcirculation [26], at the same time has little effect on the normal regional arterial circulation [27], [28]. Endovascular therapy in the acute management of ischemic stroke has become more common [29].

Takabatake et al. [30] found that the combination therapy with edaravone and sodium ozagre in the acute ischemic stroke group was significantly higher than that in the normal control group. The finding supports our conclusion. In the study by Zhang et al., [6] their investigation stated that ozagrel is effective for the improvement of neurological impairment for AIS patients. Feng et al. [31]showed that an effective treatment trend of edaravone for acute ischemic stroke. So to speak, these methods are proven useful for the acute ischemic stroke. The present meta-analyses did not reveal which one is the best choice in the treatment of acute ischemic stroke. However, through a review of the past literature and network meta-analysis, the optimal way was extracted to treat acute ischemic stroke in this several commonly used treatment. According to our study, systematic literature review was performed in order to identify RCTs of therapy options for symptomatic, previously untreated AIS. For all outcomes, the network meta-analysis confirmed that edaravone combined with Kininogenase were superior to placebo. While several other treatment strategies were significantly better than placebo for some end points, none of the other first-line treatment strategies (Sodium ozagrel, Sodium ozagrel combined with edaravone and edaravone) was significantly better than edaravone combined with Kininogenase for AIS. In four out of ten between-drug comparisons, edaravone combined with Kininogenase was significantly better than other treatment for AIS outcomes. This network meta-analysis provides compelling evidence that edaravone combined with Kininogenase was the most effective first-line treatment for AIS morbidity.

The NIHSS score strongly predicts the likelihood of a patient's recovery after stroke [32]. Our results also showed that the baseline NIHSS was significantly related to the functional outcome at 14 days in patents with AIS. After adjustment for this effect, gathered results still indicated that Sodium ozagrel combined with edaravone significantly improved the functional outcome. MISHINA et al. [33] reports of Sodium ozagrel combined with edaravone have not indicated serious adverse reaction, Sodium ozagrel combined with edaravone in treatment of cerebral infarction, which may be a promising therapeutic option. These results are consistent with those of previous view.

The concept of combination therapy in the treatment of acute ischemic stroke, Toni et al., has been proposed in 1997 [34]. The paper suggest that the effect of drug combination than alone therapy in treatment of acute ischemic stroke is better [35].It has been hypothesized that no single drug will have maximal efficacy and that a cocktail approach may be needed to promote neuroprotection [36]. Thus, success may come with methods that enable screening of combination therapies. A combination therapy strategy may provide synergy of effects. It is even likely that multiple pleiotropic drugs will be combined to treat stroke [37].

Until these trials are reported, however, our network meta-analysis provides a useful and complete picture. This technique not only includes the results from all clinical trials that directly compare two initial treatment of acute cerebral stroke in short-term clinical trials, but also incorporates indirect comparisons (particularly important for edaravone versus Sodium ozagrel combined with edaravone or Sodium ozagrel versus edaravone combined with Kininogenase, which have not yet been directly compared), and results in estimates that are highly coherent and robust to many sensitivity analyses. These attributes make this technique very attractive for summarizing the disparate results of many different clinical trials.

In recent years, network meta-analysis has become one of the focuses of people's attention. This method requires further proof. More and more studies which were carried out using a Bayesian were published in the authoritative journals, such as LANCET, STROKE, and Cochrane library and so on. However, compared with traditional Meta-analysis, network Meta-analysis the least understood but most important research method.

## Supporting Information

Figure S1 **Search results and selection of papers.**
(TIFF)

Figure S2 **Results of network meta-analysis of indirectly comparison.** E, edaravone; O, sodium ozagrel; O+E, sodium ozagrel combined with edaravone; E+K, edaravone combined with Kininogenase; CI: confidence intervals; RR, relative risk.
(TIF)

Figure S3 **Network of clinical trials of the treatment of acute cerebral stroke in which AIS was reported.** For each pair-wise comparison, arrowhead points to class of treatment of acute cerebral stroke in short-term clinical trials with acute ischemic stroke in traditional meta-analyses. Summary odds ratio and 95% CI for comparison are shown below arrow. * means indirect comparisons.
(TIFF)

Figure S4 **NIHSS WMD.** WMD: weighted mean difference; CI: confidence intervals.
(TIF)

Figure S5 **Inconsistency for triangular loops.** O, Sodium ozagrel; E, Edaravone; O+E, Sodium ozagrel combined with edaravone E+K, Edaravone combined with Kininogenase.
(TIF)

Table S1 **Characteristics of included trials of treatment for acute ischemic stroke.**
(DOC)

Table S2 **Methodological quality of included trials.**
(XLS)

Checklist S1 **PRISMA Checklist of this meta-analysis.**
(DOC)
