# Supplementary figures and images for: Efficacy and Safety of Therapies for Acute Ischemic Stroke in China: A Network Meta-Analysis of 13289 Patients from 145 Randomized Controlled Trials
Source: PLoS One. 2014 Feb 13;9(2):e88440. doi: 10.1371/journal.pone.0088440 (PMC3923787; doi:10.1371/journal.pone.0088440)

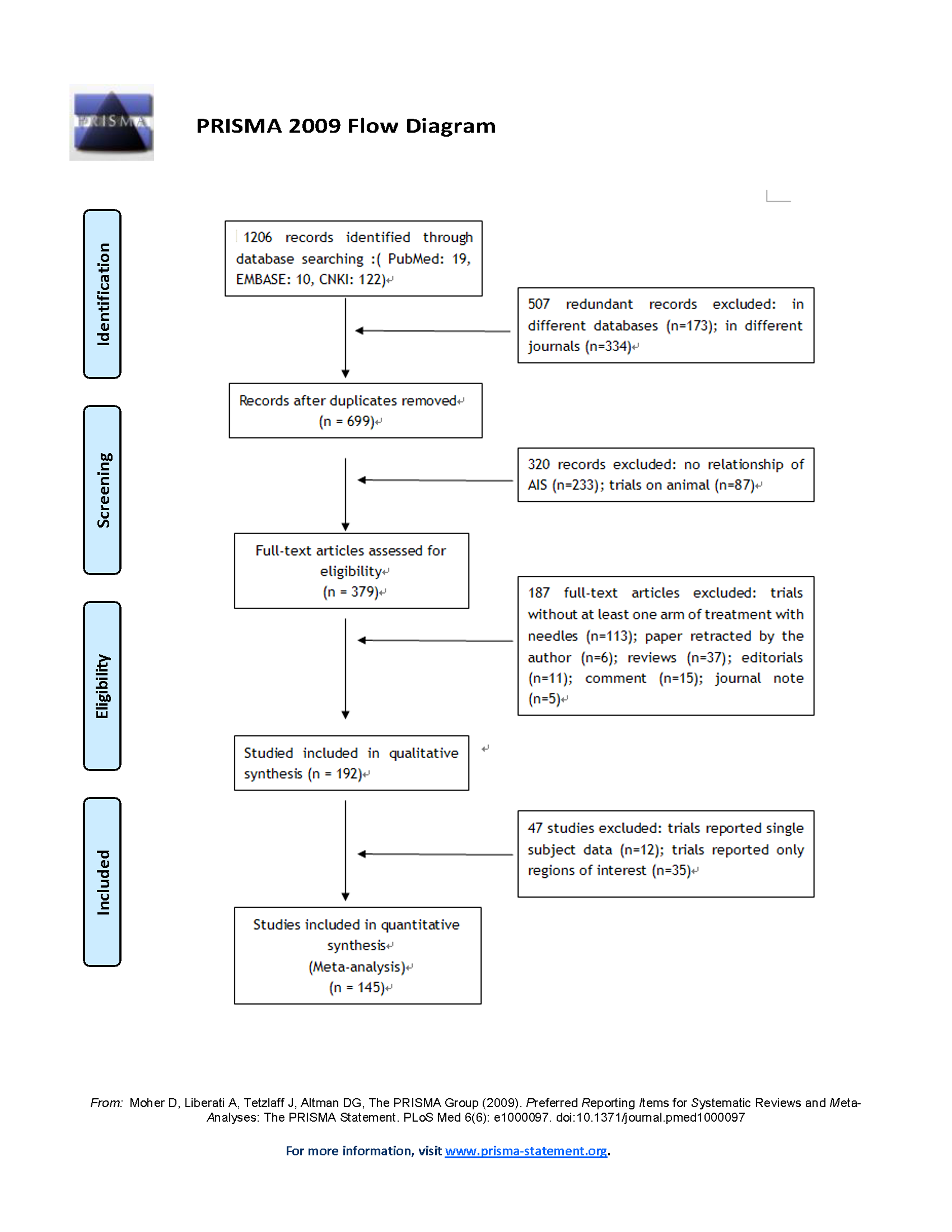

Supplement: Figure S1 — Search results and selection of papers. (TIFF) [file pone.0088440.s001.tif]

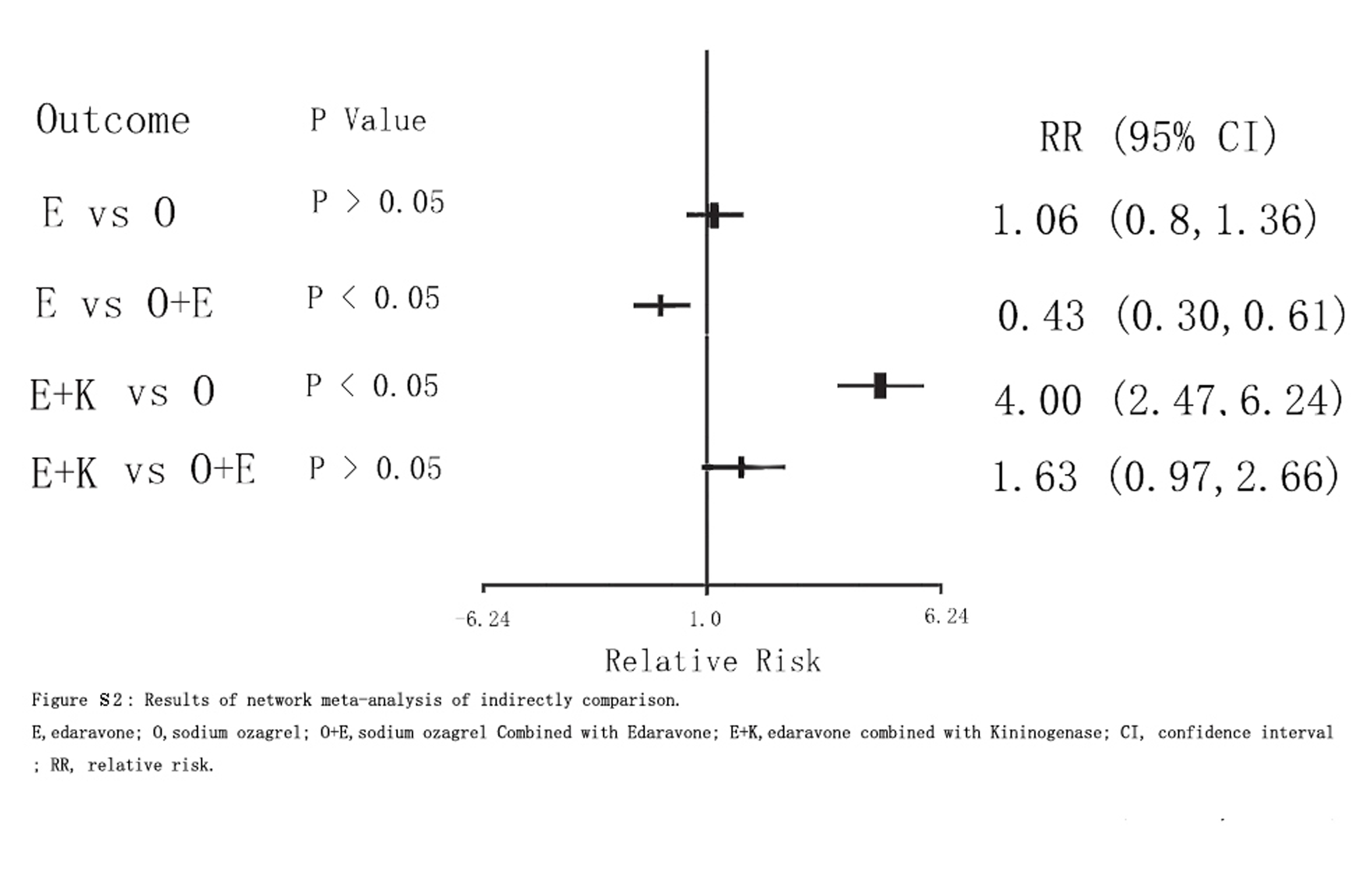

Supplement: Figure S2 — Results of network meta-analysis of indirectly comparison. E, edaravone; O, sodium ozagrel; O+E, sodium ozagrel combined with edaravone; E+K, edaravone combined with Kininogenase; CI: confidence intervals; RR, relative risk. (TIF) [file pone.0088440.s002.tif]

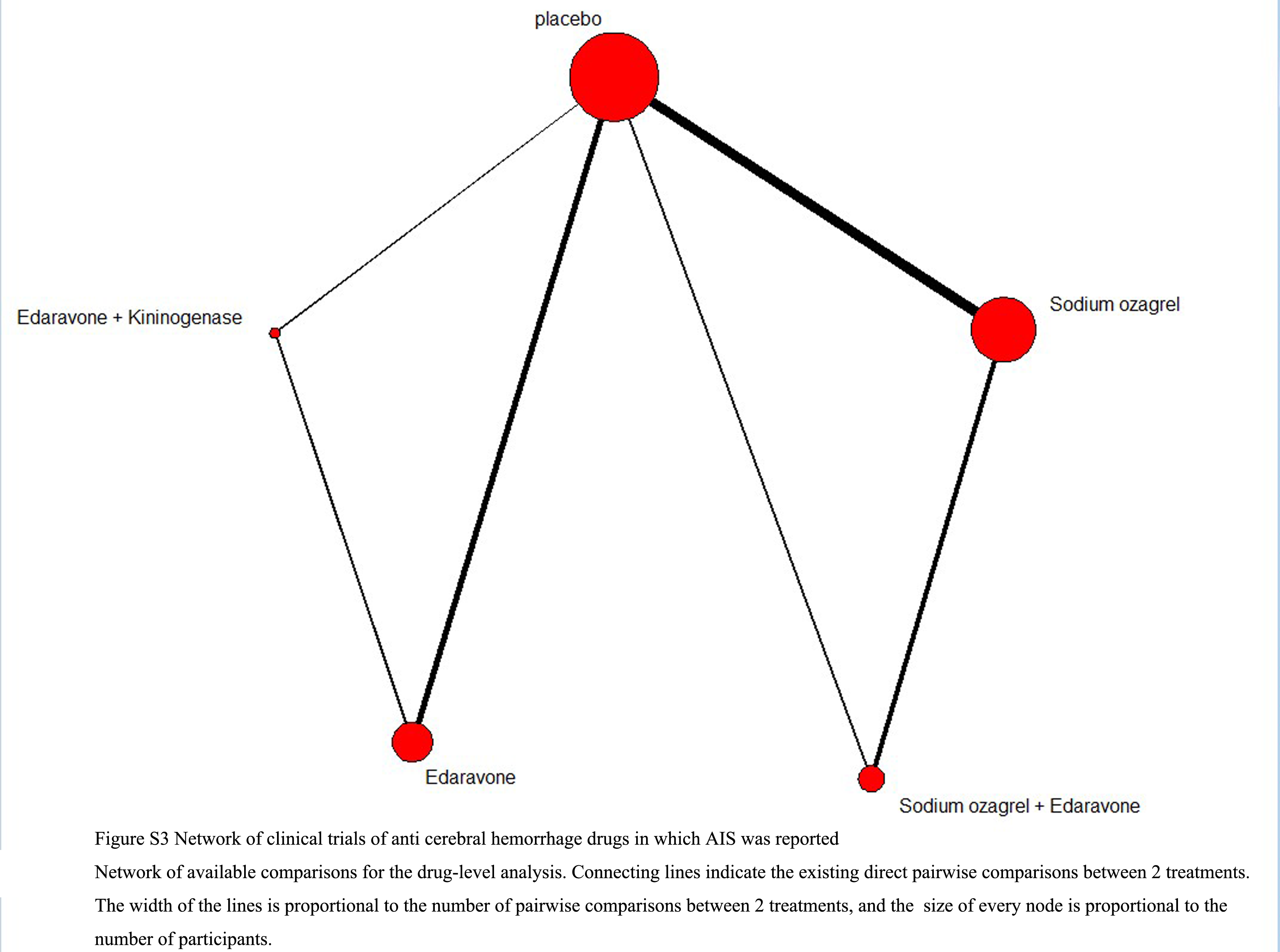

Supplement: Figure S3 — Network of clinical trials of the treatment of acute cerebral stroke in which AIS was reported. For each pair-wise comparison, arrowhead points to class of treatment of acute cerebral stroke in short-term clinical trials with acute ischemic stroke in traditional meta-analyses. Summary odds ratio and 95% CI for comparison are shown below arrow. * means indirect comparisons. (TIFF) [file pone.0088440.s003.tif]

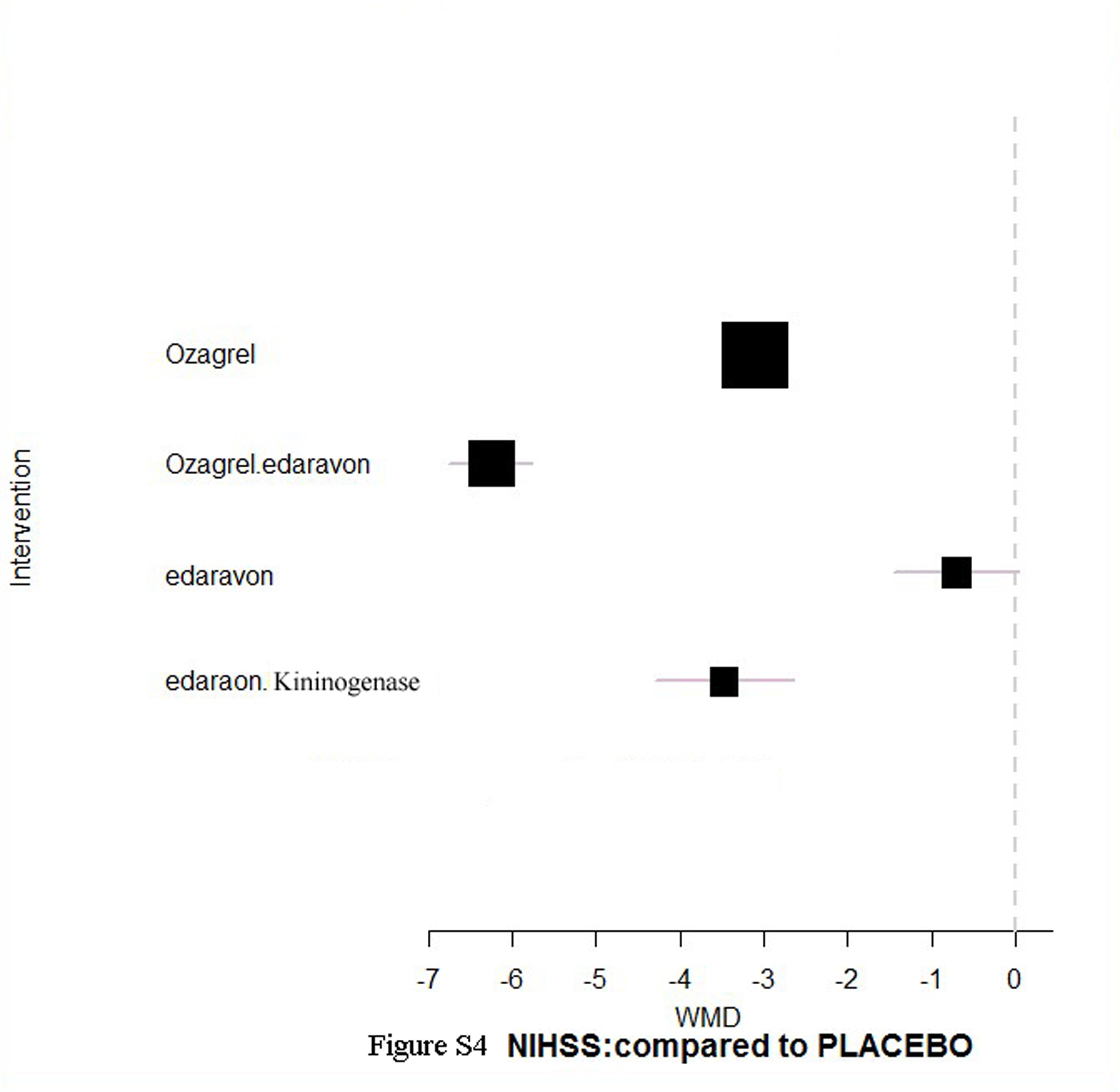

Supplement: Figure S4 — NIHSS WMD. WMD: weighted mean difference; CI: confidence intervals. (TIF) [file pone.0088440.s004.tif]

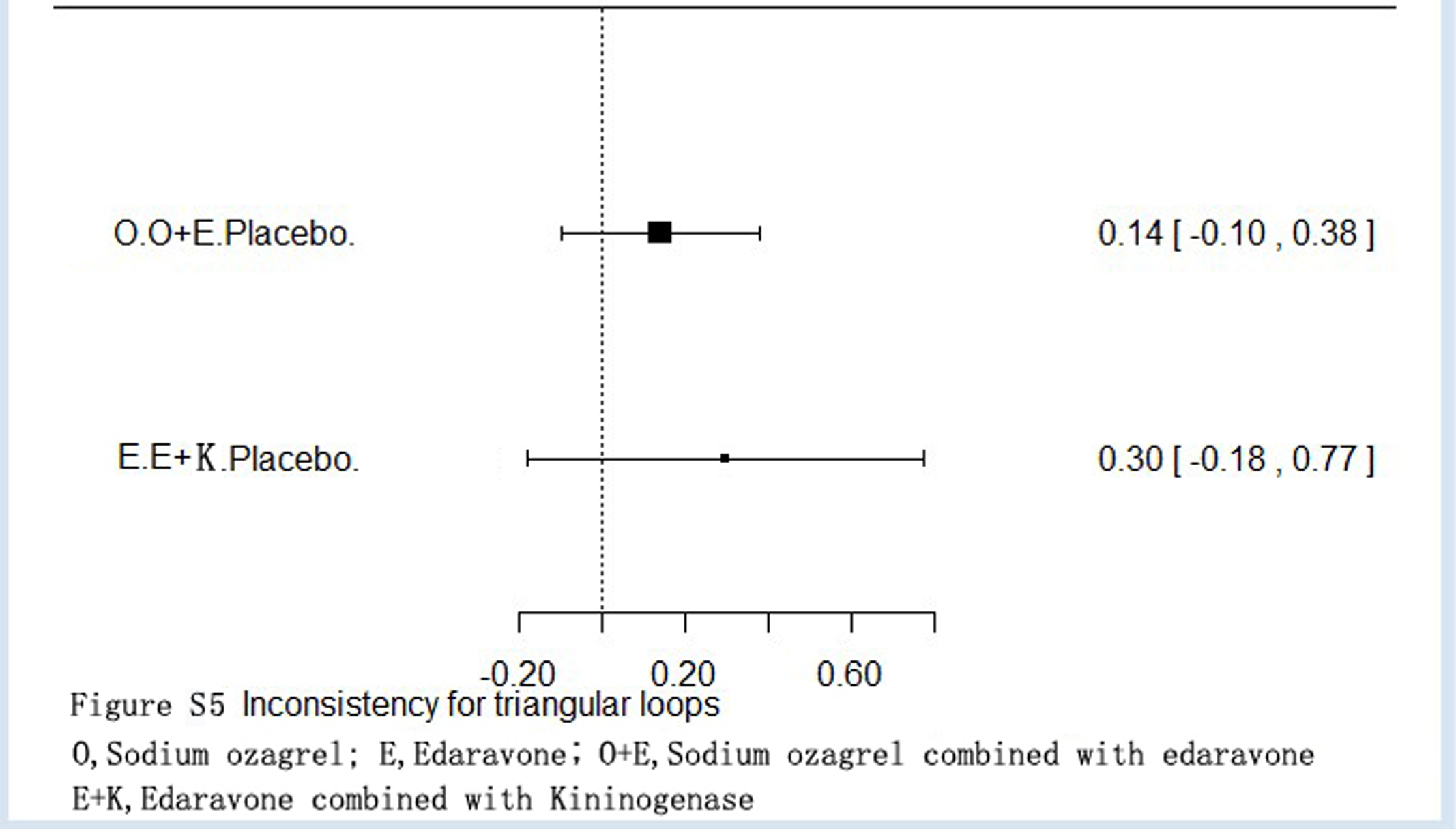

Supplement: Figure S5 — Inconsistency for triangular loops. O, Sodium ozagrel; E, Edaravone; O+E, Sodium ozagrel combined with edaravone E+K, Edaravone combined with Kininogenase. (TIF) [file pone.0088440.s005.tif]
