# Supplementary material for: Efficacy and Safety of Therapies for Acute Ischemic Stroke in China: A Network Meta-Analysis of 13289 Patients from 145 Randomized Controlled Trials
Source: PLoS One. 2014 Feb 13;9(2):e88440. doi: 10.1371/journal.pone.0088440 (PMC3923787; doi:10.1371/journal.pone.0088440)
Supplement: Table S1 — Characteristics of included trials of treatment for acute ischemic stroke. (DOC) [file pone.0088440.s006.doc]

| Table S1 Characteristics of included trials of treatment for acute ischemic stroke | | | | | | | | | | | | |  | |
| --- | --- | --- | --- | --- | --- | --- | --- | --- | --- | --- | --- | --- | --- | --- |
| Studies | Participants | | Placebo | Ozagrel | Duration of treatment (day) | | Blinding | % of  females | The scores of neurological deficit | | Drug dosage (mg) | |  | |
|  | N | Mean Ages |  |  |  |  |  |  | Placebo | Ozagrel | Placebo | Ozagrel |  | |
| Zhao et al.(2005) | 120 | 61.5 | 55/60 | 58/60 | 14 | | Double‐blind | 34.17 | - | - | + | II 80^2^ |  | |
| Liao et al.(2009) | 68 | 62.4 | 18/29 | 37/39 | 14 | | Double‐blind | 38.54 | 55. 32±1. 64 | 54. 64±1. 45 | + | II 80^2^ |  | |
| Zhao et al.(2010) | 168 | 68.3 | 66/84 | 74/84 | 14 | | Single‐blind | 35.21 | 23·56±9·92 | 23·62±10·01 | + | II 80^2^ |  | |
| Yu et al.(2009) | 86 | 69.2 | 36/43 | 40/43 | 14 | | Unclear | 48.84 | 20.5±7.3 | 21.3±8.5 | + | II 80^2^ |  | |
| Zhang et al.(2005) | 99 | 72 | 45/49 | 34/50 | 14 | | Double‐blind | 48.48 | - | - | + | II 80^2^ |  | |
| Lin et al.(2003) | 62 | 67.2 | 25/30 | 31/32 | 14 | | Double‐blind | 43.55 | - | - | + | II 80^2^ |  | |
| Mo (2010) | 70 | 61.3 | 30/35 | 34/35 | 14 | | No | 34.29 | - | - | + | II 80^2^ |  | |
| An (2008) | 76 | 63.5 | 24/36 | 38/40 | 14 | | Double‐blind | 46.05 | - | - | + | II 80^2^ |  | |
| Ji (2006) | 80 | 68 | 27/40 | 37/40 | 14 | | Single‐blind | 30 | - | - | + | II 80^1^ |  | |
| Yu et al.(2007) | 82 | 62.3 | 31/40 | 40/42 | 14 | | Double‐blind | ns | - | - | + | II 80^2^ |  | |
| Wu (2006) | 72 | 63.1 | 22/30 | 39/42 | 14 | | Double‐blind | 41.67 | - | - | + | II 80^2^ |  | |
| Wang et al.(2004) | 85 | 65.3 | 36/42 | 40/43 | 15 | | Double‐blind | 44.71 | 17.96±8.86 | 18.18±7.48 | + | II 80^1^ |  | |
| Wei et al.(2006) | 90 | 64.2 | 35/45 | 41/45 | 14 | | Double‐blind | 26.67 | 22.7±9.27 | 23.1±9.15 | + | II 80^2^ |  | |
| Zhang (2009) | 77 | 69.2 | 24/32 | 42/45 | 14 | | Single‐blind | 41.56 | - | - | + | II 80^2^ |  | |
| Long (2011) | 96 | 63.4 | 40/48 | 46/48 | 14 | | Double‐blind | 39.58 | - | - | + | II 80^2^ |  | |
| Liu (2003) | 100 | 71.4 | 30/50 | 48/50 | 14 | | No | ns | 21.58±10.59 | 21.69±11.43 | + | II 80^2^ |  | |
| Huang (2009) | 100 | 59.1 | 40/50 | 48/50 | 14 | | Single‐blind | 33 | - | - | + | II 80^1^ |  | |
| Zhao (2009) | 100 | 56.3 | 36/50 | 48/50 | 14 | | Unclear | 46 | - | - | + | II 80^2^ |  | |
| Yu et al.(2005) | 90 | 68.2 | 34/40 | 46/50 | 15 | | Double‐blind | 46.67 | - | - | + | II 80^2^ |  | |
| Lou et al.(2004) | 120 | 63.3 | 45/60 | 58/60 | 14 | | Double‐blind | 45.83 | 23.56±6.14 | 25.07±6.32 | + | II 80^1^ |  | |
| Zhang (2012) | 108 | 64.1 | 35/48 | 56/60 | 14 | | Single‐blind | 33.33 | - | - | + | II 80^1^ |  | |
| Wang et al.(2007) | 129 | 62.3 | 56/65 | 55/64 | 14 | | No | 47.27 | 21.8±6.9 | 22.1±6.8 | + | II 80^2^ |  | |
| Wu et al.(2010) | 66 | 64.3 | 23/33 | 30/33 | 14 | | Double‐blind | 42.42 | 35.1±5.4 | 32.6±6.1 | + | II 80^2^ |  | |
| Chen et al.(2008) | 158 | 61.2 | 64/78 | 76/80 | 14 | | Double‐blind | 44.49 | - | - | + | II 80^2^ |  | |
| Zhang et al.(2011) | 80 | 68.2 | 29/40 | 37/40 | 14 | | Double‐blind | 42.5 | - | - | + | II 80^2^ |  | |
| Guan (2010) | 179 | 63.5 | 59/89 | 87/90 | 14 | | No | 30.17 | - | - | + | II 80^2^ |  | |
| Fu (2000) | 64 | 62.9 | 16/32 | 22/32 | 14 | | Double‐blind | 40.63 | 28±12 | 26±14 | + | II 80^2^ |  | |
| Zhao et al.(2007) | 78 | 71.3 | 12/37 | 32/41 | 14 | | No | 46.15 | 16±7 | 16±7 | + | II 80^2^ |  | |
| Huang et al.(2005) | 100 | 63.5 | 40/50 | 46/50 | 14 | | Double‐blind | 34.74 | 25.9±4.6 | 24.4±3.5 | + | II 80^1^ |  | |
| Li et al.(2010) | 100 | 63.2 | 41/50 | 46/50 | 14 | | Single‐blind | 31 | - | - | + | II 80^2^ |  | |
| Li et al.(2004) | 80 | 68.2 | 26/40 | 38/40 | 14 | | No | 28.75 | 21.8 ± 9.06 | 22.1 ± 9.54 | + | II 80^1^ |  | |
| Liu et al.(2006) | 58 | 65.3 | 20/28 | 27/30 | 14 | | Double‐blind | 41.38 | 31.2 | 32.4 | + | II 80^2^ |  | |
| Liu et al.(2009) | 120 | 67.2 | 48/60 | 56/60 | 14 | | Single‐blind | 35.83 | - | - | + | II 80^2^ |  | |
| Zhang et al.(2009) | 90 | 63.2 | 31/44 | 42/46 | 14 | | No | Ns | - | - | + | II 80^1^ |  | |
| Wang et al.(2005) | 160 | 59.2 | 52/80 | 74/80 | 14 | | Double‐blind | 36.25 | 24.23±3.60 | 24.13±3.83 | + | II 80^2^ |  | |
| Wang et al.(2008) | 160 | 49.3 | 61/80 | 73/80 | 14 | | No | 44.38 | 20.2±9.70 | 21.1±9.04 | + | II 80^2^ |  | |
| Wang et al.(2005) | 95 | 56.3 | 35/47 | 46/48 | 14 | | Unclear | 44.21 | - | - | + | II 80^2^ |  | |
| Wei et al.(2006) | 123 | 68.3 | 52/61 | 59/62 | 14 | | No | 40.65 | 21.9±7.6 | 22.3±8.0 | + | II 80^2^ |  | |
| Zhao (2006) | 80 | 67.2 | 32/40 | 38/40 | 14 | | Double‐blind | 22.5 | 20. 06±10. 11 | 24. 32±9. 54 | + | II 80^2^ |  | |
| Che (2010) | 60 | 61.3 | 24/30 | 28/30 | 14 | | No | 45 | 24. 05 ±5. 18 | 24. 08 ± 5. 16 | + | II 80^2^ |  | |
| Huang et al.(2008) | 86 | 62.5 | 39/44 | 41/42 | 14 | | Double‐blind | 30.23 | - | - | + | II 80^2^ |  | |
| Li et al.(2009) | 178 | 63.5 | 79/87 | 90/91 | 14 | | Double‐blind | 33.14 | 23. 68 ± 10. 18 | 22. 47 ± 9. 2 | + | II 80^2^ |  | |
| Xu et al.(2000) | 83 | 63.9 | 30/41 | 39/42 | 14 | | Single‐blind | 38.55 | 22. 7 ± 10. 8 | 22. 4 ± 11. 4 | + | II 80^2^ |  | |
| Fan (2010) | 110 | 62.8 | 32/54 | 49/56 | 14 | | Single‐blind | 47.27 | - | - | + | II 80^2^ |  | |
| Xie et al.(2003) | 52 | 63.6 | 10/26 | 23/26 | 14 | | No | 38.46 | 27.19±7.11 | 27.45±6.23 | + | II 80^2^ |  | |
| Li et al.(2009) | 76 | 76.2 | 31/37 | 35/39 | 14 | | Double‐blind | 46.25 | 18·19±8·20 | 19·50±7·03 | + | II 80^2^ |  | |
| Liao et al.(2010) | 120 | 64.5 | 42/60 | 56/60 | 14 | | Double‐blind | 43.33 | - | - | + | II 80^2^ |  | |
| Zhou et al.(2005) | 80 | 69.2 | 22/40 | 34/40 | 14 | | Unclear | 42.5 | - | - | + | II 80^2^ |  | |
| Zhang et al.(2010) | 80 | 63.8 | 29/40 | 38/40 | 14 | | No | 45 | - | - | + | II 80^2^ |  | |
| Lin et al.(2004) | 60 | 63.1 | 22/30 | 28/30 | 14 | | Single‐blind | ns | - | - | + | II 80^2^ |  | |
| Peng et al.(2007) | 120 | 63.5 | 46/60 | 58/60 | 14 | | Double‐blind | 31.67 | - | - | + | II 80^2^ |  | |
| Sun et al.(2005) | 113 | 63.9 | 42/52 | 58/61 | 14 | | Double‐blind | 29.20 | - | - | + | II 80^2^ |  | |
| Chang et al.(2010) | 72 | 62.5 | 18/36 | 30/36 | 14 | | No | 44.44 | 23 ± 6 | 24 ± 5 | + | II 80^2^ |  | |
| Wang et al.(2008) | 100 | 63.3 | 41/50 | 48/50 | 14 | | Single‐blind | 46 | - | - | + | II 80^2^ |  | |
| Pu et al.(2012) | 80 | 62.5 | 29/40 | 36/40 | 14 | | Double‐blind | 41.25 | - | - | + | II 80^2^ |  | |
| Peng et al.(2011) | 92 | 63.9 | 33/46 | 42/46 | 14 | | Double‐blind | 33.7 | - | - | + | II 80^2^ |  | |
| Yu et al.(2009) | 80 | 67.3 | 22/40 | 35/40 | 14 | | Double‐blind | 38.75 | - | - | + | II 80^1^ |  | |
| Total N | 5511 |  |  |  |  |  |  |  |  |  |  |  |  | |
| Studies | Participants | | Ozagrel | Ozagrel+edaravone | Duration of treatment (day) | | Blinding | % of  females  Placebo | The scores of neurological deficit | | Drug dosage (mg) | |  |  |
|  | N | Mean Ages |  |  |  |  |  |  | Ozagrel | Ozagrel+edaravone | Ozagrel | Ozagrel+edaravone |  | |
| Chen et al. (2008) | 80 | 65.2 | 28/40 | 39/40 | 14 | | Single‐blind | 42.5 | 11±7 | 5±3 | II 80^2^ | II 80^2^+II 30^2^ |  | |
| Bai et al.(2009) | 40 | 63.3 | 14/18 | 21/22 | 14 | | Unclear | 41.26 | 14.5±5.8 | 8.4±6.2 | II 80^2^ | II 80^2^+II 30^2^ |  | |
| Fan et al.(2008) | 78 | 68.3 | 27/38 | 38/40 | 14 | | No | 40.23 | - | - | II 80^2^ | II 80^2^+II 30^2^ |  | |
| Guo et al.(2009) | 93 | 69.4 | 33/45 | 46/48 | 14 | | Double‐blind | 49.21 | 12±5 | 8±3 | II 80^2^ | II 80^2^+II 30^2^ |  | |
| Chen et al.(2006) | 70 | 75.1 | 27/35 | 33/35 | 30 | | Double‐blind | 33.21 | - | - | II 80^2^ | II 80^2^+II 30^2^ |  | |
| Wang et al.(2008) | 60 | 61.5 | 22/30 | 27/30 | 14 | | Double‐blind | 35.6 | 19.56±11.06 | 13.46±10.26 | II 80^2^ | II 80^2^+II 30^2^ |  | |
| Dou et al.(2008) | 88 | 60.3 | 34/44 | 41/44 | 14 | | Double‐blind | 37.21 | 17.45±8.07 | 15.73±7.81 | II 80^2^ | II 80^2^+II 30^2^ |  | |
| Chen et al.(2008) | 62 | 60.7 | 20/32 | 26/30 | 14 | | Double‐blind | 31.62 | 15±7 | 10±6 | II 80^2^ | II 80^2^+II 30^2^ |  | |
| Wen et al.(2006) | 80 | 63.5 | 21/40 | 35/40 | 14 | | Single‐blind | 38.2 | - | - | II 80^2^ | II 80^2^+II 30^2^ |  | |
| Wang (2009) | 83 | 63.9 | 31/42 | 37/41 | 14 | | No | 45.6 | - | - | II 80^2^ | II 80^2^+II 30^2^ |  | |
| Qu et al.(2009) | 90 | 62.8 | 34/45 | 41/45 | 14 | | Double‐blind | 49.2 | 14.43±6.05 | 10.2±5.75 | II 80^2^ | II 80^2^+II 30^2^ |  | |
| Li et al.(2008) | 94 | 60.2 | 37/46 | 44/48 | 14 | | Double‐blind | 41.3 | 18.68±6.25 | 16.28±5.46 | II 80^2^ | II 80^2^+II 30^2^ |  | |
| Zhu et al.(2009) | 86 | 63.5 | 34/43 | 39/43 | 14 | | Unclear | 23.1 | - | - | II 80^2^ | II 80^2^+II 30^2^ |  | |
| Ma et al.(2009) | 90 | 63.1 | 37/45 | 41/45 | 14 | | No | Ns | - | - | II 80^2^ | II 80^2^+II 30^2^ |  | |
| Wu et al.(2009) | 128 | 60.3 | 31/46 | 75/82 | 14 | | Double‐blind | 22.5 | 19.61±10.69 | 14.52±11.21 | II 80^2^ | II 80^2^+II 30^2^ |  | |
| Li et al.(2008) | 69 | 63.5 | 21/34 | 29/35 | 14 | | Double‐blind | 33.6 | - | - | II 80^2^ | II 80^2^+II 30^2^ |  | |
| Huang et al.(2008) | 90 | 62.3 | 35/45 | 40/45 | 14 | | Double‐blind | 38.56 | 12.44±7.82 | 7.43±5.41 | II 80^2^ | II 80^2^+II 30^2^ |  | |
| Li et al.(2009) | 108 | 61.3 | 37/54 | 48/54 | 28 | | Single‐blind | 34.1 | 12.26±5.31 | 9.48±5.13 | II 80^2^ | II 80^2^+II 30^2^ |  | |
| Deng et al.(2008) | 84 | 62.8 | 29/42 | 36/42 | 28 | | Double‐blind | 43.32 | 13.26±3.86 | 8.11±4.2 | II 80^2^ | II 80^2^+II 30^2^ |  | |
| Wu et al.(2009) | 120 | 63.9 | 46/60 | 54/60 | 14 | | Single‐blind | 45.12 | - | - | II 80^2^ | II 80^2^+II 30^2^ |  | |
| Li et al.(2009) | 120 | 64.2 | 43/60 | 48/60 | 14 | | Double‐blind | 41.23 | - | - | II 80^2^ | II 80^2^+II 30^2^ |  | |
| Total N | 1813 |  |  |  |  |  |  |  |  |  |  |  |  | |
| Studies | Participants | | Placebo | Ozagrel+edaravone | Duration of treatment (day) | | Blinding | % of  females | The scores of neurological deficit | | Drug dosage (mg) | |  |  |
|  | N | Mean Ages |  |  |  |  |  |  |  |  |  |  |  |  |
|  |  |  |  |  |  |  |  |  | Placebo | Ozagrel+edaravone | Placebo | Ozagrel+edaravone |  |  |
| Zhou et al.(2009) | 68 | 69.2 | 24/33 | 33/35 | 14 | | Single‐blind | 42.13 | 18.62±4.38 | 14.34±3.23 | + | II 80^2^+II 30^2^ |  |  |
| Hu et al.(2009) | 70 | 63.3 | 23/35 | 32/35 | 14 | | Double‐blind | 45.3 | 17.62±4.15 | 15.28±4.29 | + | II 80^2^+II 30^2^ |  | |
| Pan et al.(2008) | 160 | 61.3 | 63/80 | 77/80 | 14 | | Double‐blind | 46.21 | - | - | + | II 80^2^+II 30^2^ |  | |
| Wang et al.(2009) | 80 | 64.3 | 20/30 | 44/50 | 10 | | No | 32.5 | 13.5±5.8 | 9.5±6.8 | + | II 80^2^+II 30^2^ |  | |
| Wang et al.(2008) | 80 | 62.5 | 31/40 | 36/40 | 14 | | Double‐blind | 38.4 | 16.45±10.34 | 10.36±9.55 | + | II 80^2^+II 30^2^ |  | |
| Bi et al.(2009) | 194 | 68.3 | 84/97 | 93/97 | 14 | | Single‐blind | 39.12 | - | - | + | II 80^2^+II 30^2^ |  | |
| Yuan et al.(2009) | 82 | 64.3 | 22/40 | 34/42 | 14 | | Double‐blind | 34.26 | 14.26±7.12 | 9.32±4.81 | + | II 80^2^+II 30^2^ |  | |
| Yan et al.(2008) | 200 | 63.2 | 82/100 | 94/100 | 14 | | Double‐blind | 31.56 | - | - | + | II 80^2^+II 30^2^ |  | |
| Yang et al.(2008) | 132 | 63.1 | 51/66 | 59/66 | 14 | | Single‐blind | 38.26 | - | - | + | II 80^2^+II 30^2^ |  | |
| Total N | 1066 |  |  |  |  | |  |  |  |  |  |  |  | |
| Studies | Participants | | Edaravone | Edaravone+ Kininogenase | Duration of treatment (day) | | Blinding | % of  females | The scores of neurological deficit | | Drug dosage (mg) | |  |  |
|  | N | Mean Ages |  |  |  |  |  |  | Edaravone | Edaravone+ Kininogenase | Edaravone | Edaravone+ Kininogenase |  | |
| Li et al.(2011) | 69 | 62.1 | 28/40 | 41/45 | 14 | | Double‐blind | 35.79 | 14·21±5·01 | 14·52±4·78 | II 30^2^ | II 0.15PNA^1^+ II 30^2^ |  | |
| Guan et al.(2010) | 60 | 63.3 | 22/30 | 26/30 | 14 | | Single‐blind | 41.67 | 18·82±7·69 | 18·81±6·72 | II 30^2^ | II 0.15PNA^1^+ II 30^2^ |  | |
| Chen et al.(2010) | 90 | 65.2 | 36/45 | 44/45 | 14 | | No | 45.56 | 21. 5 ± 9. 4 | 22. 0 ± 9. 5 | II 30^2^ | II 0.15PNA^1^+ II 30^2^ |  | |
| Yi et al.(2011) | 40 | 61.3 | 15/20 | 16/20 | 14 | | Double‐blind | ns | 8. 0 ±2. 103 | 9. 15 ± 4. 38 | II 30^2^ | II 0.15PNA^1^+ II 30^2^ |  | |
| Lin et al.(2011) | 80 | 64.3 | 32/40 | 39/40 | 14 | | Double‐blind | 33.75 | 21.36 ± 9.41 | 22.45 ± 9.45 | II 30^2^ | II 0.15PNA^1^+ II 30^2^ |  | |
| Ma et al.(2011) | 83 | 63.2 | 25/40 | 40/43 | 14 | | Unclear | 30.12 | 24.36±2.16 | 23.09±3.77 | II 30^2^ | II 0.15PNA^1^+ II 30^2^ |  | |
| Cai et al.(2011) | 122 | 63.1 | 47/61 | 56/61 | 14 | | Double‐blind | 37.70 | 8.08±4.44 | 7.7±3.34 | II 30^2^ | II 0.15PNA^1^+ II 30^2^ |  | |
| Yi et al.(2009) | 80 | 63.9 | 28/41 | 34/39 | 14 | | Single‐blind | 35 | 14.6±5.5 | 14.1±5.7 | II 30^2^ | II 0.15PNA^1^+ II 30^2^ |  | |
| Li et al.(2011) | 50 | 63.8 | 19/25 | 22/25 | 14 | | Double‐blind | 38 | - | - | II 30^2^ | II 0.15PNA^1^+ II 30^2^ |  | |
| Zhang et al.(2011) | 68 | 63.5 | 24/34 | 30/34 | 14 | | Single‐blind | 38.24 | - | - | II 30^2^ | II 0.15PNA^1^+ II 30^2^ |  | |
| He et al.(2010) | 84 | 61.3 | 24/42 | 41/42 | 14 | | No | 46.43 | 40.71±6.16 | 42.36±5.62 | II 30^2^ | II 0.15PNA^1^+ II 30^2^ |  | |
| Kou et al.(2012) | 62 | 64.3 | 18/31 | 27/31 | 14 | | Double‐blind | 43.55 | - | - | II 30^2^ | II 0.15PNA^1^+ II 30^2^ |  | |
| Lu et al.(2012) | 20 | 68.2 | 4/10 | 9/10 | 14 | | Single‐blind | 40 | 25.65±2.84 | 25.8±3.97 | II 30^2^ | II 0.15PNA^1^+ II 30^2^ |  | |
| Lin et al.(2012) | 68 | 70.2 | 24/34 | 31/34 | 14 | | Double‐blind | 38.24 | 15.54 ± 4.17 | 16.34 ± 3.27 | II 30^2^ | II 0.15PNA^1^+ II 30^2^ |  | |
| Total N | 976 |  |  |  |  | |  |  |  |  | II 30^2^ | II 0.15PNA^1^+ II 30^2^ |  | |
| Studies | Participants | | Placebo | Edaravone+ Kininogenase | Duration of treatment (day) | | Blinding | % of  females | The scores of neurological deficit | | Drug dosage (mg) | |  |  |
|  | N | Mean Ages |  |  |  |  |  |  | Placebo | Edaravone+ Kininogenase | Placebo | Edaravone+ Kininogenase |  | |
| Dang et al.(2010) | 60 | 62.9 | 19/30 | 26/30 | 14 | | Double‐blind | 45 | 26.68±7.24 | 27.42±5.69 | + | II 0.15PNA^1^+ II 30^2^ |  | |
| Lin et al.(2012) | 68 | 64.3 | 24/34 | 31/34 | 14 | | Single‐blind | 38.24 | - | - | + | II 0.15PNA^1^+ II 30^2^ |  | |
| Xi et al.(2012) | 60 | 61.5 | 20/30 | 26/30 | 14 | | Double‐blind | 41.67 | 24.70 ±6.25 | 21.46 ±7.50 | + | II 0.15PNA^1^+ II 30^2^ |  | |
| Total N | 188 |  |  |  |  | |  |  |  |  |  |  |  | |
| Studies | Participants | | Placebo | Edaravone | Duration of treatment (day) | | Blinding | % of  females | The scores of neurological deficit | | Drug dosage (mg) | |  | |
|  | N | Mean Ages |  |  |  |  |  |  | Placebo | Edaravone | Placebo | Edaravone |  | |
| Otomo (2003) | 250 | 63.2 | 12/125 | 27/125 | 14 | | Unclear | 48.16 |  |  | + | II 30^2^ |  | |
| Zhang et al.(2007) | 202 | 61.2 | 38/102 | 78/100 | 14 | | Double‐blind | 47.24 |  |  | + | II 30^2^ |  | |
| Zhou et al.(2007) | 44 | 63.6 | 13/22 | 19/22 | 14 | | Double‐blind | 47.32 |  |  | + | II 30^2^ |  | |
| Liu et al.(2009) | 80 | 62.3 | 27/40 | 36/40 | 14 | | Double‐blind | 41.23 |  |  | + | II 30^2^ |  | |
| Wu et al.(2007) | 61 | 68.2 | 21/30 | 28/31 | 14 | | Double‐blind | 41.0 |  |  | + | II 30^2^ |  | |
| Wu et al.(2006) | 120 | 69.4 | 28/60 | 43/60 | 14 | | No | 45.32 |  |  | + | II 30^2^ |  | |
| Wu et al.(2010) | 83 | 673.5 | 29/41 | 38/42 | 14 | | Double‐blind | 41.9 |  |  | + | II 30^2^ |  | |
| Yu et al.(2009) | 100 | 62.5 | 20/50 | 40/50 | 14 | | No | 43 | 27.84±8.79 | 26.78±8.31 | + | II 30^2^ |  | |
| Yao et al.(2008) | 106 | 63.1 | 32/50 | 50/56 | 14 | | Single‐blind | 48 |  |  | + | II 30^2^ |  | |
| Yao et al.(2006) | 70 | 63.9 | 20/35 | 32/35 | 14 | | Double‐blind | 33.2 |  |  | + | II 30^2^ |  | |
| Jiang et al.(2009) | 60 | 62.8 | 23/30 | 27/30 | 10 | | Double‐blind | 35 |  |  | + | II 30^2^ |  | |
| Sun et al.(2010) | 63 | 65.3 | 25/33 | 29/30 | 14 | | Single‐blind | 41.2 |  |  | + | II 30^2^ |  | |
| Shou et al.(2008) | 80 | 65.1 | 19/35 | 37/45 | 14 | | Double‐blind | 45.3 |  |  | + | II 30^2^ |  | |
| Zhang et al.(2009) | 60 | 69.2 | 23/30 | 29/30 | 14 | | Double‐blind | 32.5 |  |  | + | II 30^2^ |  | |
| Zhang et al.(2008) | 80 | 63.8 | 27/40 | 35/40 | 14 | | Single‐blind | 31.5 |  |  | + | II 30^2^ |  | |
| Qu et al.(2006) | 82 | 64.2 | 20/41 | 34/41 | 8~14 | | Double‐blind | 32.4 |  |  | + | II 30^2^ |  | |
| Li et al.(2008) | 60 | 65.3 | 22/30 | 27/30 | 14 | | Double‐blind | 3.89 |  |  | + | II 30^2^ |  | |
| Li et al.(2007) | 63 | 63.1 | 22/31 | 31/32 | 14 | | Unclear | 41.7 |  |  | + | II 30^2^ |  | |
| Li et al.(2009) | 50 | 62.3 | 7/20 | 23/30 | 14 | | Single‐blind | 30 |  |  | + | II 30^2^ |  | |
| Du et al.(2009) | 84 | 68.9 | 22/42 | 32/42 | 14 | | Double‐blind | 31.6 |  |  | + | II 30^2^ |  | |
| Ouyang et al.(2008) | 80 | 63.8 | 30/40 | 37/40 | 15 | | Single‐blind | 35.2 |  |  | + | II 30^2^ |  | |
| Shen et al.(2007) | 125 | 64.1 | 38/60 | 59/65 | 14 | | No | 45.56 |  |  | + | II 30^2^ |  | |
| Pan et al.(2010) | 60 | 62.3 | 20/30 | 25/30 | 14 | | Double‐blind | 42.32 |  |  | + | II 30^2^ |  | |
| Wang et al.(2006) | 65 | 61.3 | 26/33 | 29/32 | 10 | | Double‐blind | 41.9 |  |  | + | II 30^2^ |  | |
| Wang et al.(2007) | 86 | 63.5 | 22/40 | 40/46 | 14 | | Double‐blind | 32.6 |  |  | + | II 30^2^ |  | |
| Wang et al.(2005) | 62 | 62.7 | 17/31 | 27/31 | 14 | | Double‐blind | 38.2 |  |  | + | II 30^2^ |  | |
| Wang et al.(2006) | 60 | 63.9 | 20/30 | 27/30 | 14 | | Double‐blind | 34.7 |  |  | + | II 30^2^ |  | |
| Wang et al.(2010) | 51 | 63.5 | 19/26 | 23/25 | 14 | | Unclear | 35.1 |  |  | + | II 30^2^ |  | |
| Wang et al.(2008) | 100 | 63.4 | 33/50 | 44/50 | 14 | | Double‐blind | 32 | 57.8±16.8 | 53.4±15.1 | + | II 30^2^ |  | |
| Chen et al.(2007) | 60 | 62.5 | 16/30 | 24/30 | 14 | | No | 31.42 |  |  | + | II 30^2^ |  | |
| Su et al.(2008) | 60 | 61.3 | 17/30 | 30/30 | 14 | | Double‐blind | 48.33 | 17·8±4·5 | 17·5±4·2 | + | II 30^2^ |  | |
| Dong et al.(2010) | 32 | 68.2 | 12/16 | 15/16 | 14 | | Single‐blind | 31.23 |  |  | + | II 30^2^ |  | |
| Jia et al.(2010) | 86 | 63.8 | 28/40 | 40/46 | 14 | | Double‐blind | 34.26 |  |  | + | II 30^2^ |  | |
| Shao et al.(2006) | 60 | 61.2 | 20/30 | 24/30 | 14 | | Single‐blind | 37.54 |  |  | + | II 30^2^ |  | |
| Zhou et al.(2008) | 96 | 61.3 | 36/48 | 44/48 | 10~14 | | Unclear | 46.56 |  |  | + | II 30^2^ |  | |
| Zhong et al.(2007) | 78 | 62.9 | 29/39 | 36/39 | 14 | | Double‐blind | 48.1 |  |  | + | II 30^2^ |  | |
| Wen et al.(2010) | 80 | 64.3 | 24/40 | 35/40 | 14 | | Double‐blind | 49.25 |  |  | + | II 30^2^ |  | |
| Chen et al.(2009) | 100 | 68.2 | 28/50 | 38/50 | 10 | | Double‐blind | 44 | 14. 1±5. 2 | 13. 8±5. 7 | + | II 30^2^ |  | |
| Chen et al.(2009) | 70 | 63.9 | 22/35 | 31/35 | 14 | | Single‐blind | 45.32 |  |  | + | II 30^2^ |  | |
| Bao et al.(2009) | 124 | 64.2 | 44/62 | 56/62 | 14 | | Double‐blind | 32.51 |  |  | + | II 30^2^ |  | |
| Qi et al.(2009) | 80 | 67.1 | 29/40 | 36/40 | 14 | | Single‐blind | 41.25 |  |  | + | II 30^2^ |  | |
| Total N | 3413 |  |  |  |  |  |  |  |  |  |  |  |  | |

II: Intravenous injection; 1: once a day; 2: twice a day

1. Zhao J, Lin L, Liu J (2005) Clinical observation of 60 cases of Ozagrel in treatment of acute cerebral infarction. Journal of nervous diseases 8: 63-64.

2. Liao X, Tang Y (2009) To observe the curative effect of Ozagrel on acute cerebral infarction. Journal of clinical and Experimental Medicine 8: 2223-2223.

3. Zhao J (2010) Evaluation of the effect of sodium ozagrel in the treatment of acute cerebral infarction. Journal of Yangtze University (NATURAL SCIENCE EDITION) 3: 045.

4. Yu G, Xie Z (2011) Ozagrel sodium in the treatment of clinical analysis of 43 cases of acute cerebral infarction. Journal of Xinjiang medicine 41: 66-66.

5. Zhang X, Wen C (2005) Sodium ozagrel in treating acute cerebral infarction. Journal of Medical Forum 8: 026.

6. Lin X, Xie X (2003) Clinical observation of 32 cases with sodium ozagrel in treating acute cerebral infarction. Journal of Heilongjiang medicine 27: 753.

7. Mo J (2010) Clinical observation on 35 cases of sodium ozagrel in treating acute cerebral infarction. China Tropical Medicine 10: 1260-1261.

8. An Z (2008) Clinical observation of 40 cases of sodium ozagrel in treating acute cerebral infarction. Chinese Journal of Modern Drug Appliaction 2: 71-72.

9. Ji Z (2006) Clinical observation of 40 cases of sodium ozagrel in treating acute cerebral infarction. Shandong Medical Journal 46: 64-65.

10. Yu F, Zhang Y (2007) Analysis of 42 cases of acute cerebral infarction treated with sodium ozagrel. Chinese Medicine Review 4: 65-66.

11. Wu J (2006) Clinical observation on 42 cases of sodium ozagrel in treating acute cerebral infarction. Chinese Journal of Difficult and Complicated Cases 5: 137-137.

12. Wang S, bao C, ding Y, Sun C (2004) Clinical observation of 43 cases of sodium ozagrel in treating acute cerebral infarction. Hainan Medical Journal 15: 35-36.

13. Wei X, Zhang Y (2006) Clinical observation of 45 cases of sodium ozagrel in treating acute cerebral infarcti. Journal of Practical Diagnosis and Therapy 20: 672-673.

14. Zhang D (2009) Clinical observation of 45 cases of sodium ozagrel in treating acute cerebral infarction. Journal of Chengde Medical College 26: 209-210.

15. Long Y (2011) Clinical observation of 48 cases of sodium ozagrel in treating acute cerebral infarction. Journal of Changchun University of Traditional Chinese Medicine 27.

16. Liu C (2003) Analysis of 50 cases of sodium ozagrel in treating acute cerebral infarction. Clinical Medicine 23: 58-59.

17. Huang Y (2009) Analysis of 50 cases of sodium ozagrel in treating acute cerebral infarction. Chinese Journal of Coal Industry Medicine 12: 709-710.

18. Zhao L (2009) Analysis of 50 cases of sodium ozagrel in treating acute cerebral infarction. Chinese Community Doctors 215: 15-15.

19. Yu M, Cong Z, Xu L (2005) Analysis of 50 cases of sodium ozagrel in treating acute cerebral infarction. Chinese Journal of Pest Control 21: 410-411.

20. Lou J, Bai H, Yu J, Yang X, Xu S, et al. (2005) Analysis of 60 cases of sodium ozagrel in treating acute cerebral infarction. Journal of Zhengzhou University(Medical Sciences) 40: 153-154.

21. Zhang H (2012) Analysis of 60 cases of sodium ozagrel in treating acute cerebral infarction. Applied Journal of General Practice 10.

22. Wang W, Wang G, Niu G (2007) 64 cases of acute cerebral infarction treated with sodium ozagrel. Herald of Medicine 3: 1.

23. Wu Y, Zhang X (2010) 66 cases of acute cerebral infarction treated with sodium ozagrel. China Healthcare Innovation: 26-26.

24. Chen H, Li M (2008) 80 cases of acute cerebral infarction treated with sodium ozagrel. Journal of Changchun University of Traditional Chinese Medicine 24: 328-328.

25. Zhang C (2011) Clinical observation of 80 cases with sodium ozagrel in treating acute cerebral infarction. Seek Medical 9.

26. Guan H (2010) Clinical observation of 90 cases of sodium ozagrel in treating acute cerebral infarction. Chinese Journal of Practical Nervous Diseases 13: 61-62.

27. Fu D (2000) Therapeutic effect of sodium ozagrel in treating acute cerebral infarction. Chinese Journal of Clinical Pharmacy 9: 276.

28. Zhao M, Liu L, Yuan F (2007) Analysis of therapeutic effect of sodium ozagrel in treatment of acute cerebral infarction. Chinese Remedies & Clinics 7: 634-635.

29. Huang T, Zhang C, Wang S (2005) To observe the therapeutic effect of sodium ozagrel in treating acute cerebral infarction. Chinese Journal of Practical Nervous Diseases 8: 6-7.

30. Li L (2010) To observe the therapeutic effect of sodium ozagrel in treating acute cerebral infarction. Modern Chinese Doctor: 59-60.

31. Li Q (2004) To observe the therapeutic effect of sodium ozagrel in treating acute cerebral infarction. Henan Journal of Practical Nervous Diseases 7: 25-26.

32. Liu E, Zhu L (2006) To observe the therapeutic effect of sodium ozagrel in treating acute cerebral infarction. The Medical Journal of Industrial Enter 19: 48-49.

33. Liu X (2009) To observe the therapeutic effect of sodium ozagrel in treating acute cerebral infarction. Modern Chinese Doctor 4: 096.

34. Zhang L, Sun Q (2009) To observe the therapeutic effect of sodium ozagrel in treating acute cerebral infarction. Neural Injury and Functional Reconstruction ISTIC 4.

35. Wang H, Wang L (2005) To observe the therapeutic effect of sodium ozagrel in treating acute cerebral infarction. Journal of Henan University of Science and Technology 23.

36. Wang X (2008) To observe the therapeutic effect of sodium ozagrel in treating acute cerebral infarction. Chinese Journal of Practical Nervous Diseases 11: 52-53.

37. Wang X, Zhang C, Zhang Q (2005) To observe the therapeutic effect of sodium ozagrel in treating acute cerebral infarction. Qilu Pharmaceutical Affairs 9.

38. Wei F, Xu L, Mao L (2006) To observe the therapeutic effect of sodium ozagrel in treating acute cerebral infarction. Strait Pharmaceutical Journal 18: 103-104.

39. Zhao L (2007) To observe the therapeutic effect of sodium ozagrel in treating acute cerebral infarction. Journal of Public Health and Preventive Medicine 18: 86-86.

40. Che J (2011) Clinical analysis of sodium ozagrel in treatment of acute cerebral infarction. Jilin Medical Journal 32: 1961-1961.

41. Huang G, He H (2008) Clinical observation of Ozagrel Sodium in the treatment of acute cerebral infarction. Lingnan Journal of Emergency Medicine 13: 367-368.

42. Li J, Han C, Fu J (2009) Clinical observation of Ozagrel Sodium in the treatment of acute cerebral infarction. Anhui Medical and Pharmaceutical Journal 13: 425-427.

43. Xu Y (2001) Clinical observation of Ozagrel Sodium in the treatment of acute cerebral infarction. Chinese Journal of Hospital Pharmacy 21: 484-485.

44. Fan Y (2010) Clinical efficacy of sodium ozagrel in treating acute cerebral infarction. Clinical Medicine: 50-51.

45. Xie J, Zhao J (2003) Analysis of clinical efficacy of sodium ozagrel in treatment of acute cerebral infarction. Chinese Journal of Critical Care Medicine 10: 041.

46. Li H, San J, Zhang R, Chen J (2009) To observe the clinical effect of sodium ozagrel in treatment of acute cerebral infarction. Heilongjiang Medicine Journal 22: 530-531.

47. Liao Y, Li W (2010) To observe the clinical effect of sodium ozagrel in treatment of acute cerebral infarction. China Tropical Medicine 10: 1255-1255.

48. Zhaou Z, Liu J, Zhou G (2005) To observe the clinical effect of sodium ozagrel in treatment of acute cerebral infarction. The theory and practice of Medicine 18: 39-40.

49. Zhang X (2009) Analysis of the clinical effect of sodium ozagrel in treatment of acute cerebral infarction. Chinese Journal of Ethnomedicine and Ethnopharmacy 18.

50. Lin X, Zhang F, Li Z (2004) Observation of Ozagrel Sodium in the treatment of acute cerebral infarction. Chinese Journal of Postgraduates of Medicine 27: 38-38.

51. Peng W (2007) Observation of Ozagrel Sodium in the treatment of acute cerebral infarction. Journal of Clinical and Experimental Medicine 6: 96-97.

52. Sun X, Liu F, Yang J (2005) Observation of Ozagrel Sodium in the treatment of acute cerebral infarction. Chinese Journal of Practical Meicine 32: 42-42.

53. Chang Y, Gao M (2010) Clinical observation on the treatment of acute cerebral infarction with ozagrel sodium. Chinese Journal of Integrative Medicine on Cardio-/Cerebrovascular Disease 8: 497-498.

54. Wang G, Sun B (2008) Clinical observation on the treatment of acute cerebral infarction with ozagrel sodium [J]. Chinese Journal of Practical Nervous Diseases 11: 93-93.

55. Pu S (2013) To observe the clinical efficacy of sodium ozagrel in treatment of acute cerebral infarction. China Health Industry 10: 61-61.

56. Peng H (2011) Analysis of Ozagrel Sodium Injection in treatment of acute cerebral infarction [J]. Medical Information 24: 3302-3303.

57. Yu L (2009) Ozagrel Sodium for injection in treatment of acute cerebral infarction clinical curative effect of 80 cases. Shanxi Medical Journal 12: 1146-1147.

58. Chen Z (2008) Edaravone combined with sodium ozagrel in the treatment of acute cerebral infarction clinical curative effect observation. Chinese Journal of Difficult and Complicated Cases 7: 416-417.

59. Bai Y, Wang W, Bai R (2009) Clinical analysis of 60 cases of progressive cerebral infarction treated with sodium ozagrel combined with edaravone. Chinese Journal of Coal Industry Medicine 12: 44-45.

60. Fan K, Xiao L (2008) 40 cases of acute cerebral infarction treated with edaravone and ozagrel sodium. The theory and practice of Medicine 21: 1404-1405.

61. Guo J, Zhu T (2009) Sodium ozagrel and edaravone in treatment of acute cerebral infarction (a report of clinical observation of 48 cases). Journal of Aerospace Medicine 20: 5-6.

62. Chen D, Dou W, Liu Y (2006) Observation of edaravone combined with sodium ozagrel in the treatment of acute cerebral infarction. Shanxi Medical Journal 35: 1027-1028.

63. Wang S (2008) Clinical observation of 30 cases of edaravone combined with sodium ozagrel in treating acute cerebral infarction. China Practical Medicine 3: 116-117.

64. Dou Z (2008) Clinical efficacy of edaravone in treatment of acute progressive cerebral infarction. Journal of Xinxiang Medical College 25: 503-504.

65. Chen Y, Wang Y, Xin L (2008) Observation of edaravone combined with ozagrel in treatment of acute cerebral infarction. Journal of Jilin Medical College 29: 130-131.

66. Wen S (2006) Effects of edaravone on acute cerebral infarction. Modern practical medicine journal 18: 787.

67. Wang X (2009) To observe the effect of edaravone combined with ozagrel in treating acute cerebral infarction. Guangdong Medical Journal 30: 1739-1740.

68. Qu X (2009) 90 cases of acute cerebral infarction treated with edaravone and ozagrel sodium. Modern Chinese Doctor: 71-71.

69. Li S (2008) Clinical study of edaravone combined with sodium ozagrel in treatment of progressive cerebral infarction. Chinese Journal of Practical Nervous Diseases 11: 9-11.

70. Zhu T, Jia J, Wang Y (2009) To observe the effect of edaravone combined with ozagrel in treating acute cerebral infarction. Chinese Journal of Modern Drug Application 3: 128-129.

71. Ma L, Ma Y (2009) To observe the effect of edaravone combined with ozagrel in treating acute cerebral infarction. Chinese Journal of Practical Nervous Diseases 12: 45-46.

72. Wu C, Shu G, Zhuang H (2009) Clinical observation on 82 cases of edaravone combined with ozagrel sodium in treatment of cerebral infarction. Practical Clinical Medicine 10: 36-37.

73. Lie B, Cui Y, Lin Y, Sun X, Zhao H, et al. (2008) Observation of edaravone combined with sodium ozagrel in the treatment of acute cerebral infarction. Pharmaceutical and Clinical Research 16.

74. Huang H (2008) Observation of edaravone combined with ozagrel in treatment of acute cerebral infarction. Medical journal of communications 22: 495-496.

75. Li D, Chen Y, Sun X (2009) Evaluation of edaravone combined with sodium ozagrel in treatment of acute cerebral infarction. China Healthcare Innovation 4.

76. Deng Z, Cao D, Zhou G (2008) 42 cases of acute cerebral infarction treated with edaravone and ozagrel sodium. Chinese Medicine Review 5: 11-12.

77. Wu L (2009) Edaravone combined with sodium ozagrel in treatment of cerebral infarction in clinical research. Chinese Journal of Practical Nervous Diseases 12: 51-52.

78. Li A, Kong L, Li J (2009) Clinical evaluation of edaravone combined with ozagrel in treating acute cerebral infarction. Clinical Medicine 29: 18-20.

79. Zhou G, Li D (2009) To observe the therapeutic effect of sodium ozagrel combined with edaravone treatment of progressive cerebral infarction. Contemporary Medicine 15: 91-92.

80. Hu Q (2009) The progress of clinical observation of treatment of cerebral infarction with ozagrel and edaravone. Public Medical Forum Magazine 13: 618-619.

81. Pan X (2008) Observation of therapeutic effect of edaravone combined with ozagrel sodium in the treatment of cerebral infarction. Chinese Journal of Modern Drug Application 2: 44-44.

82. Wang Q, Guo Y, Jiang C (2009) Observation of sodium ozagrel combined with edaravone in treatment of acute cerebral infarction. Journal of Shantou University Medical College 22: 141-142.

83. Wang W, Li A, Wang Y (2008) Observation of sodium ozagrel and edaravone treatment of acute cerebral infarction. Chinese Journal of Practical Nervous Diseases 11: 70-71.

84. Bi H (2009) To observe the effects of acupuncture needle combined with sodium ozagrel in the treatment of acute cerebral infarction. Contemporary Medicine 15: 147-148.

85. Yuan F (2009) Sodium ozagrel combined with edaravone in treatment of acute cerebral infarction. Journal of Shandong Medical College 31: 374-376.

86. Yan M, Li C (2008) Clinical observation of Edaravone and ozagrel sodium in the treatment of cerebral infarction. Journal of Chinese Modern Medicine 5: 52-53.

87. Yang C, Li D, Yue R (2008) Clinical observation of 66 cases of edaravone combined with sodium ozagrel in treating acute cerebral infarction. Shandong Medical Journal 48: 60.

88. Li D (2011) Kininogenase combined with edaravone in treatment of acute cerebral infarction after curative effect observation cycle. Chinese Journal of Practical Nervous Diseases 14: 42-43.

89. Guan W, Li J (2010) Observation of Kininogenase combined with edaravone treatment of progressive cerebral infarction. Jilin Medical Journal 31: 3217-3217.

90. Cheng Z, Li H (2012) Kininogenase combined with edaravone in treatment of acute cerebral infarction: clinical observation. China Practical Medicine 7: 149-150.

91. Yi L, Shen H, Han J, Zhou Z (2011) Clinical control study of Kininogenase combined with edaravone in treatment of acute ischemic stroke. Stroke and Nervous Diseases 18: 179-181.

92. Lin D (2011) Observation of curative effect of Kininogenase combined with edaravone in treatment of acute cerebral infarction. Shandong Medical Journal 51: 77-78.

93. Ma J, Shen D (2011) Kininogenase combined with edaravone in treatment of acute cerebral infarction. China Modern Medicine 18: 34-35.

94. Cai T (2011) Observation of curative effect of Kininogenase combined with edaravone in treatment of acute cerebral infarction. Chinese Journal of Integrative Medicine on Cardio-/Cerebrovascular Disease 9: 1328-1329.

95. Yi J, Zhou Y, Mei Y (2009) Observation of Kininogenase combined with edaravone in treatment of acute cerebral infarction. Stroke and Nervous Diseases: 255-256.

96. Li J, Zhang W, Wang W (2011) Clinical observation of Kininogenase with edaravone treatment of acute cerebral infarction. China Modern Medicine 18: 56-57.

97. Zhang J (2011) Clinical application of edaravone combined with Kininogenase in the treatment of acute cerebral infarction. Strait Pharmaceutical Journal 23: 159-160.

98. He Q, Quan Y (2010) To observe the effect of edaravone combined with Kallidinogenase in treatment of acute cerebral infarction. Progress in Modern Biomedicine.

99. Kou X (2012) Observation of edaravone combined with Kallidinogenase in the treatment of acute cerebral infarction. Chinese Journal of Practical Nervous Diseases 15: 42-43.

100. Lu X (2012) Observation of edaravone combined with Kallidinogenase in the treatment of acute cerebral infarction. Journal of Chengde Medical College 29: 34-35.

101. Lin F (2012) Analysis of Kininogenase and edaravone combined with efficacy in the treatment of acute cerebral infarction. Journal of Gannan Medical University 32: 52-53.

102. Dang L, Zhou X, Wang Y (2010) To observe the effect of edaravone combined with Kallidinogenase in treatment of acute cerebral infarction. China Tropical Medicine 10: 746-747.

103. Lin C (2012) Observation of edaravone combined with Kallidinogenase in treatment of acute cerebral infarction. Chinese Community Doctors 14: 20.

104. Xi T (2012) Effect of Kininogenase combined with edaravone in treatment of acute cerebral infarction and the dynamic effects on CRP. Shandong Medical Journal 52: 65-66.

105. Otomo E, Tohgi H, Kogure K, Hirai S, Takakura K, et al. (2003) Effect of a novel free radical scavenger, edaravone (MCI-186), on acute brain infarction-Randomized, placebo-controlled, double-blind study at multicenters. Cerebrovascular Diseases 15: 222-229.

106. Zhang M, Xu L, Deng L (2007) Multicenter randomized double blind study of edaravone injection in the treatment of acute cerebral infarction and its safety. Chinese Journal of New Drugs and Clinical Remedies 26: 105-108.

107. Zhou M, Xu L, Deng L, Lu J, Ren H, et al. (2007) Efficacy and safety evaluation of edaravone injection in treatment of acute cerebral infarction: a multicenter, double-blind, and randomized controlled clinical trial. Chinese Journal of New Drugs and Clinical Remedies 26: 105.

108. Liu K (2009) Curative effect observation of 40 cases of edaravone in treatment of acute cerebral infarction. Journal of Huaihai Medicine 27: 548-549.

109. Wu J (2007) Clinical study of edaravone in the treatment of elderly patients with acute cerebral infarction. China Foreign Medical Treatment 26.

110. Wu X, Liu T, Chen H, Chen D (2006) Effect of edaravone combined with Defibrase in treating acute cerebral infarction. Guangdong Medical Journal 27: 419-420.

111. Wu K (2010) Clinical observation of edaravone combined with naloxone in treatment of acute cerebral infarction. International Medicine & Health Guidance News 12: 316-318.

112. Yu L, Wan A, Wang M (2012) Effect of ganglioside combined with edaravone in treatment of acute cerebral infarction and its influence on inflammatory factors. Modern Preventive Medicine 39: 3182-3183.

113. Yao W, Wang Y, Sun Z (2008) Clinical observation of edaravone in the treatment of ischemic stroke. Journal of North China Coal Medical College 10: 483-484.

114. Yao D, Zhang J, Wang H, Tao X, Zhang L (2013) Observation of 30 cases of stroke unit combined with edaravone in treatment of acute cerebral infarction. Medical&Pharmaceutical Journal of Chinese People's Liberation Army 25.

115. Jiang G, Zhang C (2009) Clinical observation of urokinase combined with edaravone in treatment of progressive ischemic stroke. Modern Chinese Doctor 47: 131-132.

116. Sun L, Han L (2010) Observation of Edaravone and ozagrel in treatment of progressive ischemic stroke clinical efficacy. Journal of Shanxi Medical University 41: 150-151.

117. Shou Y (2008) To observe the curative effect of edaravone injection in the treatment of ischemic stroke. Modern Journal of Integrated Traditional Chinese and Western Medicine 17: 846-846.

118. Zhang B, Zhang X, Shi J (2009) To observe the clinical effect of edaravone combined with Puerarin Injection in the treatment of acute cerebral infarction. Heilongjiang Medicine Journal 6: 054.

119. Zhang F (2008) Edaravone in treatment of 40 cases of acute cerebral infarction. China Pharmaceuticals 17: 55-56.

120. Qu H (2006) Observation of edaravone in treatment of acute cerebral infarction. Modern Health 3.

121. Li A, Bai Y, Li J (2008) In 30 cases of progressive cerebral infarction treated with edaravone. Shaanxi Medical Journal 37: 1062-1063.

122. Li Y, Liu S (2007) To observe the curative effect of edaravone combined Batroxobin donlim treatment of progressive cerebral infarction. The Journal of Practical Medicine 23: 3432-3433.

123. Li Y, Du X, Zhao X (2009) Study of edaravone in the treatment of diabetic peripheral neuropathy. Strait Pharmaceutical Journal 21: 103-105.

124. Du F, Lin H, Huang L, Du F (2009) To observe the effect of edaravone in treatment of acute cerebral infarction in 42 cases. Journal of Youjiang Medical College For Nationalities 2: 191-192.

125. Ouyang X, Yu X, Weng Y, Wang S (2008) Effect of edaravone combined with ozagrel in treatment of progressive cerebral infarction. The Journal of Practical Medicine 24: 3758-3759.

126. Shen X, Zhao Y, Wang G (2007) Progress in the clinical curative effect of edaravone in the treatment of ischemic stroke. Hainan Medical Journal 4: 000.

127. Pan Q, Chen W (2010) To observe the efficacy of edaravone on acute cerebral infarction. International Medicine & Health Guidance News 16: 1341-1342.

128. Wang S, Liu J (2006) Clinical observation of Batroxobin ccmbined with edaravone in treatment of acute cerebral infarction. The Journal of Medical Theory and Practice 2: 004.

129. Wang S, Lai J, Liu W (2007) To observe the efficacy of edaravone on acute cerebral infarction. Hainan Medical Journal 18: 79-430.

130. Wang X, Yang Q, Wu H (2005) To observe the efficacy of edaravone on acute cerebral infarction. Modern Diagnosis & Treatment 16: 17-18.

131. Wang L, Lin D (2006) To observe the efficacy of edaravone on acute cerebral infarction. Hainan Medical Journal 17: 54-55.

132. Wang C (2010) Clinical observation of edaravone combined with buflomedil in the treatment of acute cerebral infarction. Shaanxi Medical Journal: 483-484.

133. Wang F, Zhang j (2008) Plasminogen with clinical observation on the treatment of cerebral infarction with edaravone in acute stage. Hubei Journal of Traditional Chinese Medicine 30: 16-17.

134. Chen J, Wang C, Bai X (2007) To observe the effects of treatment of 30 cases of acute cerebral infarction. Ningxia Medical Journal 29: 54-55.

135. Shu Q, Liu X, Wu J, Zhang Y, Xu S, et al. (2008) To observe the effect of early rehabilitation training on patients with Acute Cerebral Apoplexy Combined with edaravone. Prevention and Treatment of Cardio-Cerebral-Vascular Disease 8: 253-255.

136. Dong L (2010) Clinical observation of 16 cases of edaravone in the treatment of progressive cerebral infarction. Journal of Huaihai Medicine 28: 59-61.

137. Jia A (2010) Effects of edaravone on acute cerebral infarction. Chinese Medicine Review 7: 86-87.

138. Shao S (2006) To observe the clinical efficacy of edaravone in treatment of acute cerebral infarction. Journal of Community Medicine 4: 21-21.

139. Zou Q, Luo W, Luo Q (2008) To observe the curative effect of donlim pure batroxobin with edaravone treatment of progressive cerebral infarction. Chinese Journal of Primary Medicine and Pharmacy 15: 994-995.

140. Zhong H, Zhong D (2007) Observation of therapeutic effect of edaravone combined with naloxone in treatment of severe cerebral infarction. Modern Hospital 7.

141. Wen H, Yang J, Zhou H (2010) 40 cases of acute cerebral infarction with edaravone combined with Danshen Injection. Shaanxi Medical Journal: 480-481.

142. Chen H (2009) Effects of edaravone on acute cerebral infarction. Journal of Hainan Medical College 15: 1255-1255.

143. Chen Z (2009) Safflower injection and edaravone combined with clinical observation on the treatment of acute cerebral infarction. Shaanxi Medical Journal 38: 1660-1661.

144. Bao X, Zhu J, Ye J (2009) To observe the curative effect of edaravone injection and compound salvia miltiorrhiza injection in the treatment of acute cerebral infarction. Chinese Journal of Postgraduates of Medicine.

145. Qi H, Chu W, Feng X, Tang J, Zheng K (2009) Observation of therapeutic effect of edaravone combined with safflower injection in treatment of cerebral infarction. China Clinical Practical Medicine 3: 105-106.
